# How sustainable marketing influences the customer engagement and sustainable purchase intention? The moderating role of corporate social responsibility

**DOI:** 10.3389/fpsyg.2023.1128686

**Published:** 2023-03-15

**Authors:** Yanping Gong, Jun Xiao, Xiuyuan Tang, Jinglu Li

**Affiliations:** ^1^School of Business, Central South University, Changsha, China; ^2^School of Management, Hunan City University, Yiyang, China; ^3^School of Business, Hunan Women's University, Changsha, China

**Keywords:** sustainable marketing, brand image, customer engagement, corporate social responsibility, sustainable purchase intention

## Abstract

Today’s civilization faces serious challenges related to sustainability. Without the support of society, organizations can no longer continually build their enterprises. The pressure of sustainable development goals are also enhancing on companies. Thus, marketing managers place a strong emphasis on meeting the socio-ethical demands of their target audience, whether it is through cultural promotion, environmental conservation, or disaster relief initiatives. This study explores how sustainable marketing influences the customer engagement and sustainable purchase intention. For data collection, a self-administered questionnaire was distributed to 393 purchasers and potential purchasers of electric vehicles, and a structural equation model (SEM) test was conducted using Mplus 8.0 software. The results of the study showed that: First, the outcome states that sustainable marketing valuable for improving brand image. Second, brand image is good for customer engagement in the Chinese market for electric automobiles. Third, the appeal of sustainable purchasing intentions is increased by brand image. Fourth, a useful instrument for long-term purchase intentions is customer engagement. Fifth, CSR has a significant role in enhancing consumers’ intentions to make sustainable purchases. Most notably, it acts as a helpful moderator in the relationship between company image and customer engagement. Lastly, CSR also strengthens the link between company image and sustainable purchasing intentions. This research offers a theoretical framework and practical implications that sustainable marketing initiatives are an important antecedent of organizational outcomes for the electric vehicle sector in China.

## Introduction

Organizations prioritize sustainability while determining how to maintain steady corporate growth. The issue of how to carry out an organization’s economic, social, and environmental duties is raised by sustainability (Liu et al., 2019). By doing social actions, the organization in this work demonstrates economic sustainability. Additionally, sustainability is a major consideration while conducting CSR initiatives. Sustainable management is defined by an organization as environmental sustainability, such as adopting eco-friendly products or engaging in de-marketing initiatives. Organizations establish the groundwork for long-term success by engaging in a number of sustainability-related activities.

Additionally, the United Nations is exerting pressure through its sustainable development goals (Boluk et al., 2019). Reduced negative environmental consequences are another goal of sustainable development. Organizations’ marketing initiatives are centered on meeting the sociotechnical requirements of their target audiences, such as through disaster aid, environmental conservation, and cultural promotion (Boluk et al., 2019). The improvement of brand perception, expansion of business profits, and enduring success are all positively impacted by sustainability competitive advantage (Barney, 2016; Shaukat and Ming, 2022). By fostering client loyalty to the brand rather than merely generating short-term profit, sustainable activities are beneficial for long-term development (Prates et al., 2015). As a result, a company’s sustainable marketing initiatives foster a favorable brand perception and consumer attitude; as a result, the firm earns competitive advantages based on brand equity.

Cultural considerations must be included while conducting sustainable marketing campaigns in order to strengthen societal collaboration and increase customer-organization interaction through brand image (Ko et al., 2015). In modern customer management literature, the concept of client involvement has gained prominence. A brand needs a strong brand image to distinguish itself from its competitors. It serves as a brand representative, promotes the growth of customer connections, and makes it easier for customers to evaluate the brand (Cretu and Brodie, 2007). Brand image has an important role in engaging customers (Islam and Rahman, 2016). Moreover, sustainable purchase intention also becomes an imperative concern for companies (Huo et al., 2022).

Previous research on sustainable marketing has typically focused on examining the relationship between sustainable marketing on consumer outcomes such as consumer loyalty, satisfaction, and purchase intentions in the luxury and fast fashion industries (e.g., Sun et al., 2014; Jung et al., 2020; Kong et al., 2021). However, we select the electric vehicle market from China. Because, the energy consumption from the transportation industry has increased significantly as a result of the expansion in the Chinese automobile market (Ou et al., 2020). China’s annual gross crude oil imports in 2017 totaled more than 8.4 million barrels per day (MMb/d), compared to the US’s 7.9 MMb/d (Barron, 2020). Consequently, the market for electric cars is regarded as a trustworthy solution to problems with energy and pollution in the transportation industry (Hu et al., 2021). Because power can be generated from a variety of sources, including coal, nuclear, natural gas, and renewable sources, electric cars assist to lessen this threat (Ou et al., 2020). The Chinese government is encouraging people to buy electric automobiles only for environmental preservation, which is driving increasing demand for them (Ou et al., 2020). It highly needs to study this sector in the context of sustainable practices. In addition, a recent study suggests that consumer perceptions of fit between product and sustainability strategies are an important prerequisite for organizational results (Gleim et al., 2023). Also, another group comparison study showed an increase in sustainability perceptions and a decrease in willingness to purchase luxury products, as sustainability communications can create discordant and conflicting associations with luxury brands (Lee and Lee, 2018). Compared to the gasoline and diesel markets, electric vehicles have a higher match with sustainable marketing strategies as a result of being more environmentally friendly and economically sustainable. Therefore, the following query is prompted by this circumstance: If companies’ sustainable marketing strategies achieve a competitive edge by using brand image and customer in engagement the electric vehicle market? Furthermore, how sustainable purchase intentions could be enhanced with the help of brand image and customer engagement?

Moreover, corporate social responsibility (CSR) gives companies the chance to align their goals with social progress, which ultimately contributes to the long-term viability of the company (Luo and Bhattacharya, 2018). In addition, when consumers engage in CSR activities and are aware of them, their decision-making is affected. CSR initiatives assist consumers in making wise buying decisions (Waheed et al., 2019). Additionally, CSR policies encourage customers to make sustainable purchasing decisions. By proving that they can satisfy the conflicting needs of their stakeholders, companies that care about CSR will increase their reputation and integrity. On the one hand, CSR may have a direct impact on sustainable purchases; on the other hand, CSR is used as a moderator for checking connections amid brand image and sustainable purchase intentions and customer engagement and sustainable purchase intention. Theoretically, this study is supported by the resource-based view and stakeholder theory.

Therefore, the aim of this study is to identify the effects of sustainable marketing on sustainable purchase intentions in the electric vehicle market. For this purpose, this study uses brand image mediator through customer engagement for sustainable purchase intentions. Moreover, this study applies corporate social responsibility as a moderator of the relationship between brand image and customer engagement, and between brand image and sustainable purchase intentions.

This study is an important addition to the narrow literature on sustainable marketing practices in the context of the electric vehicle market. This study offers multiple policy guidelines to governments, stakeholders, and markers. Moreover, this study also encourages managers to promote the role of sustainable marketing for improving brand image customer, engagement, and sustainable purchase intentions.

## Theoretical framework and hypotheses

The resource-based view (RBV), a management concept, is used to identify the strategic resources a corporation might deploy to gain a sustained competitive advantage. According to the resource-based perspective theory, the foundation of long-lasting competitive advantage is valued, uncommon, and imitable resources and skills (Barney, 2016). The capacity of a business to obtain and maintain a competitive advantage is the emphasis of the steady-state view. The ability of a firm to adapt to and benefit from a dynamic environment is the focus of the dynamic capabilities perspective. Therefore, the concept of sustainable marketing is getting more important with time.

According to dynamic capabilities, firms compete not only in their capacity to utilize their current assets and organizational capabilities but also in their capacity to update and develop those organizational capabilities in order to adapt to the uncertain environment (Teece and Pisano, 1994). This theory supports sustainable marketing for enhancing intangible asset as brand image. Generally, sustainable marketing helps companies for creating a competitive advantage (Sun et al., 2014). Sustainable marketing shed light on corporate environmental and social goals which automatically enhance the brand reputation in the eyes of stakeholders (Jung et al., 2020).

Customer engagement refers to a psychological process that simulates the underlying processes by which customer loyalty builds for new consumers of a service brand as well as the methods by which loyalty may be maintained for repeat purchase customers of a service brand (Hollebeek, 2011). The branding creates a psychological image for customers which motivate their engagement. The sustainable practices of the firms improve the brand ranking and engage the customers for the long term. Brand image and the engagement of customers are really valuable for companies. Therefore, the resource-based view pushes companies for enhancing sustainable marketing for achieving competitive advantage. Sustainable marketing also provides a corporate social image which is really helpful for brand image and customer engagement.

Companies are under pressure to behave sustainably due to growing environmental and environment-related concerns. One way to achieve this is by implementing sustainable management systems or employing sustainable branding and marketing (Sun et al., 2014). Consumers are also prompted by environmental concerns to take more initiative and be receptive to green consumption. Similarly, the RBV theory helps the customers and brand for sustainable purchase intentions. Companies with a resource, or a combination of resources, that is uncommon among rivals are said to have a comparative advantage.

Due to their comparative advantage, businesses can create marketing offers that are (a) seen to have a higher value or (b) produced more cheaply. Sustainable purchase intention is numerous studies demonstrate the beneficial effects of CSR policies on customer behavior results, including product evaluation (Abdeen et al., 2016; Abbas et al., 2018; Huo et al., 2022). Compared to some of the least socially responsible companies, customers respond favorably to organizations that engage in socially responsible operations (Husnain and Toor, 2017). In this context, the stakeholder theory supports corporate social actions such as sustainable marketing, purchase intention, and relation with customers (Zhang and Ahmad, 2021). Customers are one of the key stakeholder types that respond to an organization’s CSR initiatives (Bhattacharya and Sen, 2003). The stakeholder theory is important for finding out the connection of customers with corporate social actions. Although it is unclear how customer CSR knowledge affects purchasing decisions, an organization’s CSR policies may have an influence on the welfare of communities and the mental health of its customers. CSR is defined as, “a strategy for integrating social, environmental, and consumer issues into the operations of the corporation to optimize the sharing of value for its owners and its other stakeholders, as well as for society as a whole” (Fernández-Guadaño and Sarria-Pedroza, 2018).

### Sustainable marketing and brand image

Economic, social, and environmental aspects should all be considered in sustainable marketing initiatives (Elkington, 1998). In terms of manufacturing and sales, as well as the social environment and environmental ethics of the local community and customers, sustainable marketing refers to these activities. The active discussion has resulted from the conception of sustainability in a number of domains (Ko et al., 2015). Companies utilize sustainability management efforts to reach out to customers as a societal interest. Recent initiatives in sustainable marketing have given way to shared value management that goes beyond corporate social responsibility (Sheehy and Farneti, 2021). Additionally, sustainable marketing initiatives aim to promote the growth and harmony of several variables, including the economy, environment, society, and culture (Jung et al., 2020; Gleim et al., 2023).

In addition to commercial operations targeted at generating profits, sustainable marketing efforts also help a corporation achieve other goals (Min Kong and Ko, 2017). Social interactions are also beneficial to consumers’ perceptions of a brand. Customers are kept connected to their behavioral goals, such as their responses and attitudes toward a firm’s products, through the social activities a company engages in with them (Park et al., 2017). Companies understand their social obligations as members of society and uphold their social commitments to the neighborhood. From a long-term standpoint, businesses might then develop into social enterprises. For instance, social contribution activities can involve giving out food to neighbors, offering free health checks to locals, or engaging in other volunteer work in the neighborhood. Sustainable marketing initiatives should consider social activities across the board and not just one aspect of the triple bottom line (TBL) (Ko et al., 2015).

In this sense, brand image refers to the symbolic significance connected to particular brand qualities. The belief, ideas, and impressions that a person has about a certain thing are combined to form what is known as the consumer’s cognitive image (Cretu and Brodie, 2007). According to Alexander et al. (2014), a strong brand reputation aids in positioning, improves performance, and safeguards brands from competitors. Through customer involvement, a strong brand image is likely to build consumer loyalty (Islam and Rahman, 2016). As a result, corporate CSR initiatives may significantly increase competitive advantage by enhancing brand perception and fostering favorable consumer behavior (Huo et al., 2022). Several studies have shown that corporate sustainability marketing initiatives improve the perception of the company’s brand (e.g., Jung et al., 2020; Gleim et al., 2023).

For instance, Maignan and Ferrell (2001) assert that brand development, company profit growth, and lifespan are all positively impacted by economic sustainability marketing initiatives. A company’s reputation is better when it is judged to brilliantly exercise social responsibility than when it does not (Alexander et al., 2014), as a result, people develop positive brand attitudes. Consumers will benefit from these initiatives, value the firm, and raise corporate consciousness if a company makes an investment in strengthening (Morales, 2005). In order to implement green management, environmental sustainability marketing initiatives educate local community members as well as businesses about environmental preservation (Boluk et al., 2019). Culturally appropriate marketing initiatives that take diversity into account can improve brand recognition and foster cultural peace (Min Kong and Ko, 2017; Almeida and Coelho, 2018). Hence, this study proposes the following hypothesis,

*H1*: Sustainable Marketing is beneficial for Brand Image.

### Brand image and customer engagement

Since the 1950s, brand image has generated a lot of intriguing conversation in the marketing field (Cho et al., 2015). In order to set a brand apart from its rivals, it must have a strong brand image. It represents the brand, enables the development of consumer relationships, and facilitates customer evaluation of the brand (Ramesh et al., 2019). As a result, many businesses continue to place a high importance on building their brand (Cho et al., 2015). The symbolic, practical, emotional, and rational brand beliefs make up brand image (Low and Lamb, 2000). Brand image represents one’s self-image as a representation of a brand (Sirgy, 1985). When a brand’s image matches the customer’s self-concept, customers are more likely to form stronger emotional attachments with that brand (Sirgy, 1985).

According to the majority of scholars, customer engagement (CE) is characterized by a multidimensional model comprising cognitive, emotional, and behavioral dimensions (Abbas et al., 2018). It is based on the creation of value by creating close relationships between the company and the customer. As CE involves cognitive, emotional, and behavioral responses, we believe that it is a psychological state, which motivates customers to adopt behaviors supportive of the company. Brand image really matters for customer engagement. In this setting, the literature includes some empirical research on the interactions between brand image and consumer engagement (Hollebeek, 2011). Van Doorn et al. (2010) offer an empirical analysis of the link between brand attributes and consumer engagement. Customers are more likely to be involved with a business when it is perceived as attractive and helps them feel better about themselves (Godfrey et al., 2009). In addition, earlier research has found that customers are more engaged with fashion brands when they believe they have a positive image of their organization (Islam and Rahman, 2016). Thus, this study posits the hypothesis as,

*H2*: Brand Image can be helpful for Customer Engagement.

### Brand image and sustainable purchase intention

Brand image is viewed as “the extra value with which a certain brand endows a product”(Cho et al., 2015), and marketing activities contribute to this incremental value (Keller, 2013). Because it contains characteristics that set it apart from competing items that meet the same needs, a brand is more than just a product. A brand’s strength comes from the consumer’s knowledge and experience accumulated through time (Keller, 2013). Because they offer protection from rivals (such as patents and trademarks) and may result in long-term competitive advantages.

There is considerable evidence that traditional brands’ brand image positively influences purchase intentions either directly or indirectly (Ramesh et al., 2019). Although sustainability and green marketing are significant challenges, research on green branding is less. However, brand image does have a beneficial influence on consumer attitudes and behaviors toward the brand (Moradi et al., 2011; Wu and Wang, 2014). For example, Jung et al. (2020) revealed that the enhancement of brand image caused by corporate sustainability marketing activities affects customer satisfaction, customer trust, and customer loyalty. Therefore, it is assumed in this study that brand image would serve as a meaningful predictor of green purchase intent. The primary outcome that would be impacted by sustainable marketing efforts is defined in this study as EV’s sustainable purchase intention.

*H3*: Brand Image positively influences sustainable purchase intention.

### Customer engagement and sustainable purchase intention

Previous research has shown that psychological states can motivate individuals to engage in a variety of behaviors (Müller-Stewens et al., 2017). Depending on how specific customers perceive the characteristics of CSR activities, the influence of social attributes on customers’ behavioral intentions varies (Ahn, 2020). Customers who experience a high level of autonomy and relatedness through social initiatives, for instance, are more likely to return and recommend the service provider to others. For example, according to Eisingerich et al. (2019), attributes can influence a customer’s engagement by encouraging social interaction, goals, progress tracking, rewards, and prompts.

The establishment of a deep bond between a company and its clients can be roughly characterized as consumer engagement (Glavee-Geo et al., 2019). Consumer engagement describes consumer actions that are directed toward certain businesses or brands for reasons other than simple consumption. It has to do with clients developing close emotional ties to the brand (Abbas et al., 2018). Therefore, it is possible to think of consumer engagement as a multifaceted notion that encompasses cognitive, emotional, and/or behavioral aspects. These dimensions are crucial for the creation of long-lasting connections that are advantageous for both enterprises and their clients (Hollebeek et al., 2014). Consumer engagement has been linked in the past to usage intention and loyalty (Husnain and Toor, 2017).

Additionally, prior research has shown that brand engagement has a favorable effect on consumers’ intentions to use a brand’s products (Park and Jiang, 2020). Purchase intention benefits from both a direct and indirect increase in customer involvement (Huerta-Álvarez et al., 2020). Not to add, prior research has underlined that stakeholders tend to reward businesses that engage in excellent CSR by supporting them and punish businesses that solely focus on profit without engaging in CSR activities. This is further demonstrated by the existence of a strong positive correlation between customer purchasing behavior and business social responsibility activities (Yeo et al., 2018). We will thus carefully evaluate how customer interaction affects the intention to make sustainable purchases. This study posits the hypothesis as,

*H4*: Customer engagement and sustainable purchase intention are positively connected.

### Corporate social responsibility and sustainable purchase intention

From Friedman’s traditional strategy of maximizing shareholders’ returns to the contemporary perspective of CSR as perceived societal responsibilities, or at the very least, stakeholder commitments, CSR has always been characterized in the literature from many angles (Fernández-Guadaño and Sarria-Pedroza, 2018). A business practice known as CSR is one that is connected to the stakeholders or social obligations that the company has been given (Luo and Bhattacharya, 2018). CSR enables businesses to harmonize their objectives with societal advancement, which ultimately aids in the long-term sustainability of the enterprise (Luo and Bhattacharya, 2018). Carroll and Shabana (2010) asserted that competitive edge arguments suggest that a CSR-conscious organization will build good partnerships with its customers and gain their assistance in the form of consumers’ willingness to pay a premium price, and thus achieve customer satisfaction by putting forth CSR practices.

Customers’ purchase intentions are influenced by CSR practices when they participate in and are aware of them, according to research (Abdeen et al., 2016). When a company practices CSR, consumers will think more highly of its products. CSR activities help the consumer to make good purchase decisions (Zhang and Ahmad, 2021). The world is facing environmental glitches which leading to global warming (Javeed et al., 2022). Therefore, sustainable purchase intention becomes an integral part of customers as well (Huo et al., 2022). In this setting, the CSR practices encourage the customer for sustainable purchase intention. Businesses that are concerned about CSR will gain credibility and integrity by demonstrating that they can meet the competing requirements of their stakeholders. By incorporating moral and ethical principles into their strategic decision-making, they will increase the trust of all parties involved, including customers (Hosmer, 2007). This study suggests the following hypothesis as,

*H5*: Corporate social responsibility and sustainable purchase intention are positively connected.

### Moderating effects of corporate social responsibility

The term “CSR” refers to business discretionary actions and donations for social welfare and community well-being (Perrini, 2006). Customer involvement includes any voluntary manifestation of a client’s informational, physical, behavioral, and emotional input to the client’s service process outside of just consumption (Uzkurt, 2010). Particularly, consumer participation in CSR is essential to the company’s CSR, including socially responsible behaviors and direct connections between clients and service providers (Cha et al., 2015). Existing research has demonstrated that customers’ behavior is significantly influenced by how they see the company’s CSR initiatives (Bhattacharya and Sen, 2003).

Customers’ CSR involvement is positively correlated with their favorable opinion of the company, which encourages the company to devote more resources to CSR programs (Bhattacharya and Sen, 2003). Customers’ impressions of a company’s commitment to social responsibility, for instance, have a favorable impact on customers’ intentions to make purchases and show loyalty (Bhattacharya and Sen, 2003). The company’s CSR initiatives have an impact on how customers perceive its brand and goods, as well as how they choose and promote it. This immediately increases customer engagement (Bhattacharya and Sen, 2003). Customers’ engagement in a company’s CSR has the most influence on it, as evidenced by factors like the desire to support it, fortitude in the face of unfavorable publicity, and consumer knowledge of its CSR goals. Thus, this study posits the following hypothesis as,

*H6*: Corporate social responsibility positively moderates the connection between customer engagement and sustainable purchase intention.

Both directly and implicitly, CSR has been shown to affect consumer brand reactions (Abbas et al., 2018; Huo et al., 2022). This argument proves that CSR affects how consumers feel about a brand. One of the most prevalent communication fallacies relates to brand image and how consumers see brands as represented in firm associations in the public mind (Keller, 2013). A strong brand image is advantageous because it influences consumers’ expectations for interactions with company operations (Kim and Ma, 2014). While all business initiatives will influence how consumers perceive brands, CSR is particularly influential (Lion et al., 2016). Higher revenues, a better brand image, enhanced customer loyalty, and increased market value are all positive outcomes of CSR (Abdeen et al., 2016; Abbas et al., 2018; Huo et al., 2022).

Through CSR initiatives, customers may be more firmly engaged with the company (Wu and Wang, 2014) and may develop a favorable perception of the brand among consumers (Alexander et al., 2014) In a similar vein, Brock et al. (2014) asserted that the economic, financial, and stakeholder initiatives or programs of CSR have a favorable effect on customers’ choice of brand. Deegan (2002) has shown that brand perceptions of CSR significantly contribute to brand identity and trustworthiness. Additionally, Ramesh et al. (2019) discovered a significant correlation between brand image and CSR initiatives aiming at raising social consciousness. According to Mohammed and Rashid (2018), CSR initiatives are the organization’s capacity to identify its goods and services *via* the development of a strong brand identity that aids in maintaining excellent recognition. Thus, this study posits the following hypothesis as,

*H7*: Corporate social responsibility positively moderates the connection between brand image and sustainable purchase intention.

## Research method

### Data collection and procedure

**Figure 1:**
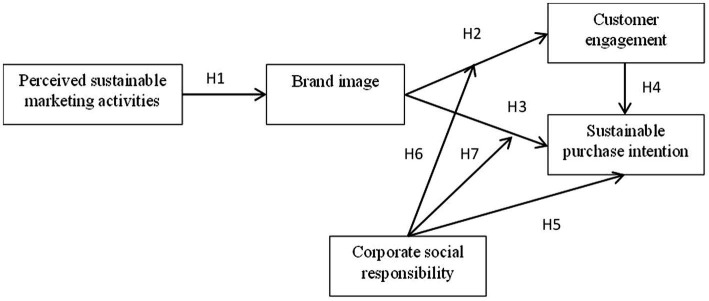
Research model.

Based on the previous discussion, the conceptual framework was drawn, as shown in Figure 1. In this study, we adopted a questionnaire survey in September–December 2022 to collect data to test the hypothesis. Referring to previous studies by Xu et al. (2020), this survey was conducted in Shanghai, Changsha, and Hefei, the first pilot cities in China for electric vehicles. In these places, EVs are more accessible, consumers get more chances to use and experience EVs, and they are more familiar with the brands available. We randomly selected eight EV stores in these three cities and distributed questionnaires to EV buyers and potential buyers by intercepting them. As a sample strategy, convenience sampling is the best appropriate option under specific population conditions to collect representative samples (Jager et al., 2017). If they had purchased an EV or were familiar with or interested in an EV brand, we proceeded to distribute the questionnaire to them. The response rate was 62%, and some people refused to participate due to “busy schedules” or “no time.” A total of 393 questionnaires were returned. In addition, the samples that failed the two filter questions or took less than 120 s to complete were disqualified as not filled out carefully. Ultimately, after removing 75 invalid questionnaires, a total of 318 valid samples were collected.

The questionnaire consisted of three parts: the first part is confirmatory questions and participants are excluded who had not purchased or used an electric car in the previous 6 months. The second part consists of the measurements of the variables, and the third section consists of the personal details of the participants, namely their gender, age, level of education, and income.

Among the 318 respondents, there were 193 participants females (60.7%). In terms of age groups, 45.6% of respondents were between 21 and 30 years old, 43.1% were between 31 and 40 years old, and 11.3% were above 41 years old, suggesting that young people are more accepting of EVs and that market is dominated by people between 20 and 40 years old. A total of 55 respondents had a college degree or less (17.3%), 195 respondents had a bachelor’s degree (61.3%), and a master’s degree or more (*n* = 68, 21.4%). In terms of annual income, 41 participants had an annual income below RMB 60,000 (12.9%), 17.9% between RMB 30,000 ~ RMB 60,000 (*n* = 57), 17.3% between RMB 60,000 ~ RMB 90,000 (*n* = 55), 25.8% between RMB 90,000 ~ RMB 120,000 (*n* = 82), and 26.1% of the participants had an annual income of RMB 120,000 or more (*n* = 83). Among them, 68% of the participants had already purchased an electric car (*n* = 220), while the remaining 34% had experienced an electric car (*n* = 98), had some knowledge of electric car brands, and were considering purchasing one in the future.

### Measurement

The current study includes five variables: perceived sustainable marketing activities (PSMA), brand image (BI), corporate social responsibility (CSR), customer engagement (CE), and sustainable purchasing behavior (SPB). The scales were adapted from previous research to ensure reliability and validity, and the wording of some items has been slightly revised to be more in line with current research. According to Jung et al. (2020) study, perceived sustainable marketing activities involve 12 items in four dimensions, three items each for economic, social, environmental, and cultural considerations. The measurement of the brand image refers to Wu and Wang (2014) ‘s study, which consists of 12 measurement items in three dimensions, four for functional image, symbolic image, and experiential image, respectively. Corporate social responsibility measure refers to Eisingerich et al. (2010)‘s study and contains three items. Based on the research of Kumar and Kaushik (2022), customer engagement contains 12 items, including four items related to cognitive engagement, three related to emotional engagement, and three related to behavioral engagement. Sustainable purchase behavior adapted from Xu et al. (2020) ‘s study includes three items. On a 5-point Likert scale, each item was rated from 1 “strongly disagree” to 5 “strongly agree.” Appendix Table A1 presents detailed measures of the variables.

## Data analysis and results

### Validity and reliability analysis of measurement

The reliability and validity of the measured variables were verified by reliability tests and confirmatory factor analysis. In this study, the questionnaire data were analyzed using SPSS 21.0 and Mplus 8.0. software. Specifically, we used SPSS 21.0 for reliability analysis and Mplus 8.0 software for confirmatory factor analysis and structural equation modeling to test the hypotheses.

SPSS 21.0 was used to determine the reliability of the measurement items, and construct reliability is usually assessed using Cronbach’s *α* values and composite reliability (CR) values. To test internal consistency, Cronbach’s *α* is used, and for the five variables, the CA was 0.85–0.92, which was above the level of 0.70 (Hair et al., 2010). Therefore, the reliability is good and acceptable. The indicator of CR is consistency in explaining the hidden variable across all its measures (Hair et al., 2010). If the value of CR is greater than 0.60, the intrinsic quality can be considered acceptable (Fornell and Larcker, 1981). The results of the reliability and validity analysis are shown in Table 1.

**Table 1:** Results of confirmatory factor analysis for measurement.

Construct		Standardized factor loading	Cronbach’s α	AVE	CR
Perceived sustainable marketing activities (PSMA)	Economic 1	0.749	0.91	0. 95	0. 64
	Economic 2	0.820			
	Economic 3	0.752			
	Social 1	0.745			
	Social 2	0.808			
	Social 3	0.818			
	Environmental 1	0.888			
	Environmental 2	0.818			
	Environmental 3	0.720			
	Cultural 1	0.777			
	Cultural 2	0.879			
	Cultural 3	0.803			
Brand image (BI)	Functional image 1	0.768	0.92	0.94	0.58
	Functional image 2	0.760			
	Functional image 3	0.811			
	Functional image 4	0.781			
	Symbolic image 1	0.801			
	Symbolic image 2	0.740			
	Symbolic image 3	0.741			
	Symbolic image 4	0.740			
	Experiential image 1	0.748			
	Experiential image 2	0.789			
	Experiential image 3	0.761			
	Experiential image 4	0.719			
Corporate social responsibility (CSR) Customer engagement (CE)	CSR 1	0.788	0.87	0.69	0.87
	CSR 2	0.883			
	CSR 3	0.815			
	Cognitive engagement 1	0.764	0.90	0.61	0.94
	Cognitive engagement 2	0.836			
	Cognitive engagement 3	0.764			
	Cognitive engagement 4	0.757			
	Affective engagement 1	0.734			
	Affective engagement 2	0.824			
	Affective engagement 3	0.821			
	Behavioral engagement 1	0.754			
	Behavioral engagement 2	0.798			
	Behavioral engagement 3	0.791			
Sustainable purchase behavior (SPB)	SPB 1	0.777	0.85	0.67	0.86
	SPB 2	0.822			
	SPB 3	0.853			

Based on the results of confirmatory factor analysis, the model fits well with *χ*^2^ = 118.99, *df* = 94, *p* = 0.04, *χ*^2^/*df* = 1.266, TLI = 0.987, CFI = 0.990, all above 0.90, RMSEA = 0.029. AVE values were all higher than 0.58, above the acceptable level of 0.50, which indicates good convergent validity of the hidden variables. Meanwhile, the square root of AVE is greater than the correlation coefficient between each pair of latent variables, which is shown in Table 2. Therefore, adequate reliability and validity for the evaluation model scales (Fornell and Larcker, 1981).

**Table 2:** Variable correlations and the square root of AVE.

Variable	1	2	3	4	5
PSMA	0.76				
BI	0.26**	0.84			
CSR	0.036	0.15**	0.83		
CE	0.22**	0.52**	0.13*	0.79	
CPB	0.07	0.41**	0.17**	0.43**	0.93

PSMA, perceived sustainable marketing activities; BI, brand image; CSR, corporate social responsibility; CE, customer engagement; SPB, sustainable purchase behavior. The diagonal values represent the square root of AVE. *p < 0.05 and **p < 0.01.

### Hypothesis verification result

The results of the hypothesis testing are shown in Table 3. This study examined the relationship between perceived sustainable marketing activities, brand image, customer engagement, and sustainable purchase intentions in the electric vehicle market. The correlations between the constructs initially supported four direct effects (H1, H2, H3, and H4) (Table 2). Our hypothesis about the relationship and the moderating effect of CSR was tested by SEM with maximum likelihood. Based on the hypothesized structural model, we found an acceptable fit (*χ*^2^ = 10.01, *df* = 4, p = 0.04, *χ*^2^/*df* = 2.50, TLI = 0.877, CFI = 0.959, RMSEA = 0.069). These indicators suggest that the model reached an acceptable level (Fornell and Larcker, 1981).

**Table 3:** Hypotheses results.

	Model 1			Model 2			
	Standardized coefficient	SE	*P*-value	Standardized coefficient	SE	*P*-value	
H1: PSMA → BI (+)	0.25	0.06	<0.001	0.23	0.06	<0.001	Supported
H2 BI → CE (+)	0.46	0.06	<0.001	0.43	0.06	<0.001	Supported
H3 BI →SPB (+)	0.29	0.08	<0.001	0.33	0.08	<0.001	Supported
H4 CE → SPB (+)	0.31	0.1	0.002	0.35	0.1	<0.001	Supported
H5 CSR → SPB	0.06	0.06	0.35	0.08	0.06	0.21	Not supported
H6 BI * CSR → CE (+)	0.27	0.09	0.002	0.25	0.09	0.004	Supported
H7 BI * CSR → SPB (+)	0.33	0.14	0.015	0.30	0.14	0.03	Supported

PSMA, perceived sustainable marketing activities; BI, brand image; CSR, corporate social responsibility; CE, customer engagement; SPB, sustainable purchase behavior. +, hypothesized positive effect. Model 1: without adjustment. Model 2: adjusted for gender, age, level of education, and income.

According to the structural model, the first four hypothesized relationships are supported empirically (see Table 3, Model 1). PSMA has a significant and positive impact on BI (*β* = 0.25, *p* < 0.001), supporting H1. These results support the previous empirical study by Jung et al. (2020) that consumer perceptions of sustainable marketing activities are beneficial to brand image. A significant, positive impact is observed by BI on both CE (*β* = 0.46, *p* < 0.001) and SPB (*β* = 0.29, *p* < 0.001), supporting H2 and H3, respectively. Additionally, CE contributes significantly to SPB (*β* = 0.31, *p* = 0.002), supporting H4. After adding the control variables, gender, age, education, and income, the standardized coefficients show a small change, but the results of the hypothesis test remain stable, further demonstrating the model’s robustness (see Table 3, Model 2). These findings are consistent with Kumar and Kaushik (2022) ‘s study that CBE leads to a higher brand usage intention.

**Figure 2:**
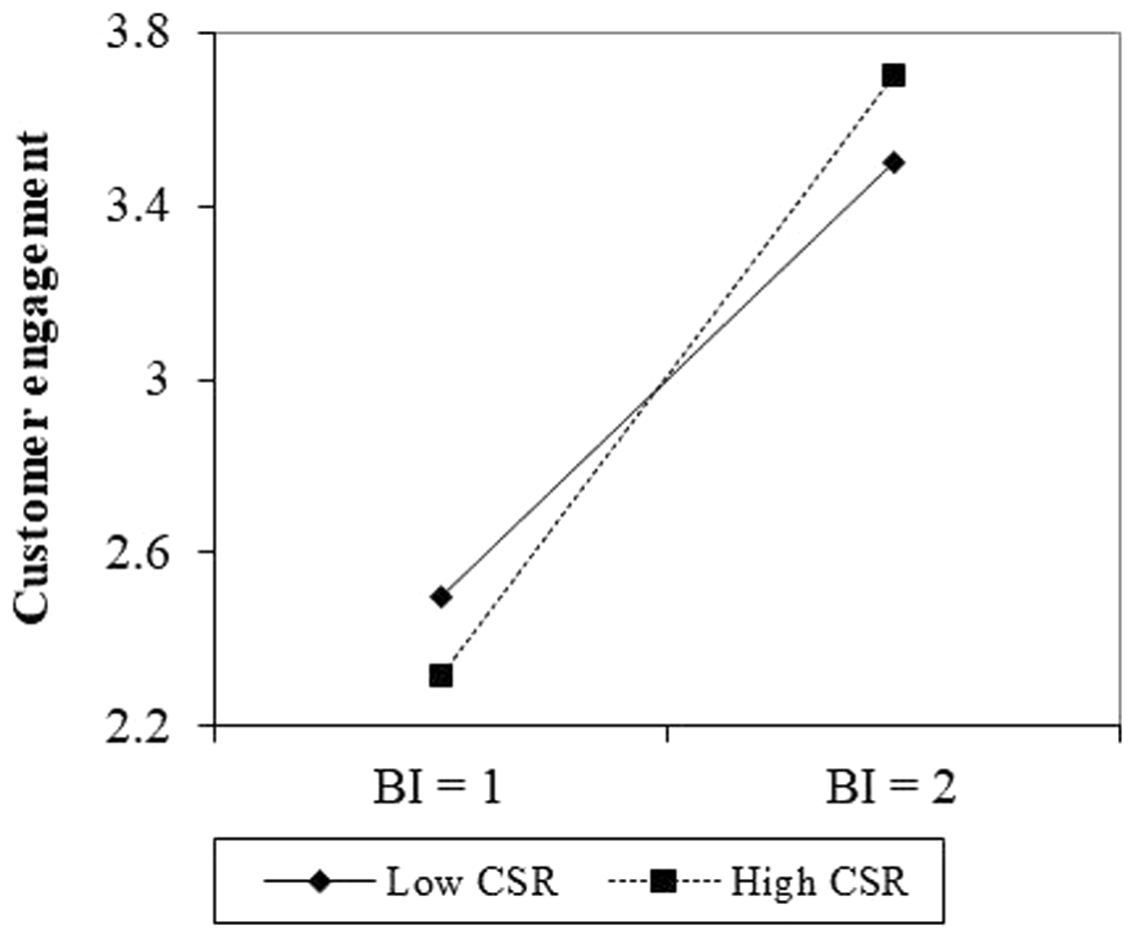
Moderating effects of corporate social responsibility on the relationship between brand image and customer engagement.

**Figure 3:**
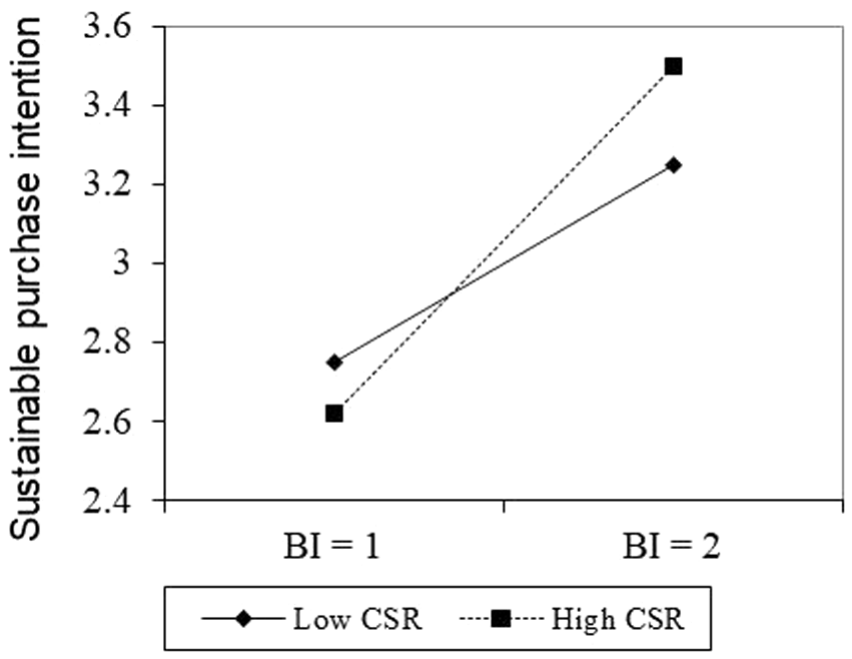
Moderating effects of corporate social responsibility on the relationship between brand image and sustainable purchase behavior.

However, the direct effect of CSR on CE (*β* = 0.01, *p* = 0.88) and PSB (*β* = 0.06, *p* = 0.35) was not significant, which indicates H5 is not supported as the hypothesized effect. Finally, after the centralization of BI and CSR, the relationship between BI and CE was positively moderated by CSR (*β* = 0.27, *p* = 0.002) at the 1% significance level. CSR also moderated the relationship between BI and PSB (*β* = 0.33, *p* = 0.015) at the 5% significance level. A plot of the interaction effects was generated at one standard deviation above and below the mean following the results described above (Slack et al., 2014). As shown in Figure 2, the positive relationship between BI and CE becomes stronger when consumers perceive the brand’s social responsibility to be higher, although BI and CE are also positively correlated when social responsibility is low. Similarly, Figure 3 shows that as a brand’s social responsibility is perceived to be higher by consumers, the positive relationship between BI and SPB becomes stronger. Therefore, H6 and H7 are empirically supported.

## Conclusion and implications

This study seeks to understand the influence of sustainable marketing through brand image, customer involvement, and corporate social responsibility in fostering consumers’ sustainable purchase intentions. After the analysis, this study provides conclusive remarks on the electric automobile market. The first conclusion is that sustainable marketing is useful for enhancing brand image. Second, brand image encourages customer engagement. Third, brand image heightens the attraction of sustainable purchase intentions. Fourth, customer engagement is an effective tool for sustainable buying intentions. Last but not least, the intention of customers to make sustainable purchases is not significantly increased by CSR. However, CSR improves the connection between brand image and customer engagement, as well as between brand image and sustainable purchase intentions.

Based on the research results, this study has several theoretical contributions. First, this study proposes the importance of sustainable marketing activities for the electric car industry and finds that they can enhance a company’s brand image and consumer engagement through sustainable marketing efforts. Specifically, the study found that sustainable marketing initiatives in the EV industry will help enhance brand image, which in turn will have a beneficial impact on customer engagement and sustainable purchasing of EVs. This discovery can serve as a springboard for expanding on related theories and the outcomes added theoretical points of view as well. This study adds to the body of academic literature already available and will aid future research on the market for electric vehicles. Second, this study confirms the relationship between brand image, customer engagement, and sustained purchases. Though they are consistent with previous studies, this study extends previous studies’ findings to electric vehicles. Third, this study shed light on the importance of CSR for improving brand image, and customer engagement relations with sustainable purchase intentions. Fourth, the findings of this study complement previous studies on green marketing. The aforementioned sustainable marketing efforts, brand image, customer engagement, and corporate social responsibility are all contributing factors that lead to customer loyalty and sustained purchase of electric vehicles.

The results of the study have significant practical implications for the promotion of electric vehicles from both corporation and government perspectives. Marketing managers must recognize the necessity of demonstrating the positive aspects of sustainable marketing. Sustainable marketing initiatives enhance brand perception, which in turn has a beneficial impact on customer engagement or purchase intentions. Therefore, firms operating in the electric car industry should focus on sustainable economic, social, environmental, and cultural practices to enhance their brand image. They will be able to endure in volatile market settings thanks to these actions. Businesses that use sustainable marketing are more productive and competitive in their primary markets.

In addition, these findings could help the government and other regulatory bodies for controlling environmental glitches caused by the traditional car market. This study promotes environmental action for sustainability, which can enhance the electric car market’s brand image. Sustainable marketing initiatives in the electric car industry will aid in creating long-lasting bonds between customers and buy intents. These actions can also boost market share and establish a competitive edge over other established markets for the regular vehicles market. The aforementioned brand image, CSR, and leveraging significant synergies from sustainable marketing efforts should become the driving forces in order to develop a strong connection based on electric car markets and consumer loyalty. These actions will probably help create a favorable perception. Importantly, the results of this study may be useful to organizations, governments, and society as a whole.

These are the study’s shortcomings and areas that future research might expand upon. The sample of the current survey consists of Chinese residents only. Due to cultural differences, generalizing the current study results to all clients in other countries requires careful consideration. Alternatively, future studies could investigate this theoretical research model in industries with similar or different profiles to further generalize the findings. Although this study reported on the demographic profile of participants (e.g., gender, age, education, and income), the mechanisms of how such demographic variables influence the research model have not been examined. Future research could investigate the impact of demographics on the relationship between study structures. Moreover, this study used a limited time period for data collection, other studies could make it longer. It is vital to do a cross-national comparison in order to assess the findings by selecting other markets as the sample.

## Data availability statement

The raw data supporting the conclusions of this article will be made available by the authors, without undue reservation.

## Ethics statement

The studies involving human participants were reviewed and approved by Central South University Institutional Review Board. The participants provided their written informed consent to participate in this study.

## Author contributions

YG contributed to the conception and design of the study, assisted with the execution of the study and data collection, and provided the critical revisions. JX contributed to the conception and design of the study, drafted the manuscript, and performed the statistical analysis. XT and JL assisted with the data collection and drafted the manuscript. All authors contributed to the manuscript reversion and approved the submitted version.

## Funding

The present research was supported by the Project of the National Natural Science Foundation of China (Grant No. 72072185).

## Conflict of interest

The authors declare that the research was conducted in the absence of any commercial or financial relationships that could be construed as a potential conflict of interest.

## Publisher’s note

All claims expressed in this article are solely those of the authors and do not necessarily represent those of their affiliated organizations, or those of the publisher, the editors and the reviewers. Any product that may be evaluated in this article, or claim that may be made by its manufacturer, is not guaranteed or endorsed by the publisher.

## Appendix

Table A1 Detailed measures of the variables.

**Table:**

Construct	Measures
Perceived sustainable marketing activities (PSMA)	*Economic*
	Efforts are made for efficient management.
	The brand makes a lot of effort for technological innovation.
	The brand improves the economic power of the electric vehicle market with various activities.
	*Social*
	The brand support activities for the community.
	The brand return some of the profits to society.
	Part of the profit is devoted to donation.
	*Environmental*
	Environmentally friendly materials used.
	Consider the environment throughout the design process.
	Prevent environmental pollution during production and distribution process.
	*Cultural*
	The brand respects various races.
	The brand recognizes international multi-culturalism.
	The brand strives for collaboration with globalization and local culture.
Brand image (BI)	*Functional image*
	The brand provides product appearance and packaging that meet consumers’ needs.
	Choosing this brand is wise.
	The brand provides excellent services.
	The brand’s EV quality is satisfactory.
	*Symbolic image*
	Enjoying the brand’s EV is trendy.
	Enjoying the brand’s EV is a symbol of social status.
	The brand’s EV is a leading brand.
	The brand’s EV and brand match my individual image.
	*Experiential image*
	The brand’s EV interest me.
	The brand’s EV services make me feel warm and comfortable.
	The brand’s EV‘s shop environment offers me enjoyment.
	The brand’s EV pursue diversified consumer needs in daily life.
Corporate social responsibility (CSR)	The brand is a socially responsible brand.
	The brand is more beneficial to society’s welfare than other brands.
	The brand contributes to society in welfare than other brands.
Customer engagement (CE)	*Cognitive engagement*
	Using the brand gets me to think about this brand.
	I think about this brand a lot when I’m using it.
	Using this brand stimulates my interest to learn more about this brand.
	I feel very positive when I use this brand.
	*Affective engagement*
	Using this brand makes me happy.
	I feel good when I use this brand.
	I’m proud to use this brand.
	Behavioral engagement
	I spent a lot of time using this brand compared with other brands.
	Whenever I’m using a product, I usually take this brand.
	This brand is one of the brands I generally use.
Sustainable purchase behavior	Compared with traditional fuel vehicles, I will give priority to buy this brand’s EVs.
	I am willing to buy this brand’s EV in the near future /have bought this brand’s EV.
	I will recommend relatives and friends around me to buy this brand’s EVs.
